# Guanine-Modified Inhibitory Oligonucleotides Efficiently Impair TLR7- and TLR9-Mediated Immune Responses of Human Immune Cells

**DOI:** 10.1371/journal.pone.0116703

**Published:** 2015-02-19

**Authors:** Franziska Römmler, Monika Hammel, Anna Waldhuber, Tina Müller, Marion Jurk, Eugen Uhlmann, Hermann Wagner, Jörg Vollmer, Thomas Miethke

**Affiliations:** 1 Institut für Medizinische Mikrobiologie, Immunologie und Hygiene, Technische Universität München, Munich, Germany; 2 Miltenyi Biotec GmbH, Bergisch Gladbach, Germany; 3 Adiutide Pharmaceuticals GmbH, Frankfurt, Germany; 4 Nexigen GmbH, Köln, Germany; 5 Institute of Medical Microbiology and Hygiene, Medical Faculty Mannheim, University of Heidelberg, Mannheim, Germany; University of Nebraska—Lincoln, UNITED STATES

## Abstract

Activation of TLR7 and TLR9 by endogenous RNA- or DNA-containing ligands, respectively, is thought to contribute to the complicated pathophysiology of systemic lupus erythematosus (SLE). These ligands induce the release of type-I interferons by plasmacytoid dendritic cells and autoreactive antibodies by B-cells, both responses being key events in perpetuating SLE. We recently described the development of inhibitory oligonucleotides (INH-ODN), which are characterized by a phosphorothioate backbone, a CC(T)XXX_3–5_GGG motif and a chemical modification of the G-quartet to avoid the formation of higher order structures via intermolecular G-tetrads. These INH-ODNs were equally or significantly more efficient to impair TLR7- and TLR9-stimulated murine B-cells, macrophages, conventional and plasmacytoid dendritic cells than the parent INH-ODN 2088, which lacks G-modification. Here, we evaluate the inhibitory/therapeutic potential of our set of G-modified INH-ODN on human immune cells. We report the novel finding that G-modified INH-ODNs efficiently inhibited the release of IFN-α by PBMC stimulated either with the TLR7-ligand oligoribonucleotide (ORN) 22075 or the TLR9-ligand CpG-ODN 2216. G-modification of INH-ODNs significantly improved inhibition of IL-6 release by PBMCs and purified human B-cells stimulated with the TLR7-ligand imiquimod or the TLR9-ligand CpG-ODN 2006. Furthermore, inhibition of B-cell activation analyzed by expression of activation markers and intracellular ATP content was significantly improved by G-modification. As observed with murine B-cells, high concentrations of INH-ODN 2088 but not of G-modified INH-ODNs stimulated IL-6 secretion by PBMCs in the absence of TLR-ligands thus limiting its blocking efficacy. In summary, G-modification of INH-ODNs improved their ability to impair TLR7- and TLR9-mediated signaling in those human immune cells which are considered as crucial in the pathophysiology of SLE.

## Introduction

Systemic lupus erythematosus (SLE) is a heterogeneous autoimmune disorder involving different organs such as skin, joints, kidneys, lung and nervous system. Although the initial events which trigger autoimmunity are unclear it was suggested that an accumulation of apoptotic and/or necrotic cells due to irregularities in the production or clearance of these cells represent the activating principle for the first wave of type I interferons [1]. This may lead to an accumulation of self-DNA and -RNA which trigger inflammation. A defective clearance of cytosolic DNA was observed in DNase II deficient mice, resulted in an IFN-β-mediated apoptosis of liver erythrocyte precursors and death in utero and points to the possibility that nucleic acids are the driving force for autoimmune inflammation [2]. These initial steps activate dendritic cells, which in turn stimulate resting autoreactive T- and B-cells to produce autoantibodies forming complexes with DNA or RNA [1,3]. The DNA- or RNA-containing complexes then activate plasmacytoid dendritic cells (pDCs) to secrete more type I interferons [4] and activate B-cells [5]. Type I interferons, thus, play a central role in this scenario and it is therefore not surprising that SLE patients display an interferogenic signature, i.e. many type I interferon induced genes are expressed [1]. These complex events lead to a self-augmenting circle of inflammation, which finally leads to organ damage and failure.

A variety of recent findings clearly point to the nucleic acid-recognizing Toll-like receptors (TLRs) to maintain the production of type I interferons. Four human and three murine TLRs recognize nucleic acids: TLR3 of both species is activated by double-stranded RNA, murine and human TLR7 and human TLR8 by single-stranded RNA and TLR9 of both species by double-stranded DNA [6]. Their involvement in SLE became apparent by the finding that disease severity in lupus-prone mouse models like the MRL-Fas^lpr^ strain was reduced by deletion of TLR7 [7]. Conversely, the Y chromosome-linked autoimmune accelerator locus in male BXSB mice contains a duplication of the TLR7 gene, which is presumably involved in the early onset of autoimmune disease in this mouse strain [8,9]. Surprisingly, TLR9 deficiency in the lupus-prone mouse strain MRL/Mp^lpr/lpr^ did not reduce but increased disease severity [7]. This unexpected finding was likely explained by the observation that TLR7 and TLR9 competed for their translocation from the endoplasmic reticulum to the endosome which was mediated by UNC93B1 [10,11]. When TLR9 was missing the chances for TLR7-translocation were higher and thus the lupus-like syndrome was aggravated. Consequently, MRL-Fas^lpr^ mice deficient for UNC93B1 showed reduced nephritis and reduced serum levels of antibodies to nuclear antigens [12]. Similarly, TLR8-deficiency led to autoimmunity with increased autoantibodies against small nuclear ribonucleoproteins and dsDNA due to an augmented expression of TLR7 and hyperresponsiveness to TLR7 ligands [13].

Endogenous ligands for TLR7 (and hTLR8) and TLR9 are RNA and DNA-complexes, respectively [4,14–16]. Thus, self-RNA and self-DNA bound to autoantibodies, the high mobility group box 1 or the antimicrobial peptide LL-37 are able to trigger immune cells, since they translocate self-RNA or -DNA across the cellular membrane into the endosomal compartment [5,17,18]. B-cells recognize DNA/RNA-antibody complexes via their surface Ig-receptors and subsequently translocate them to the endosomal compartment which induces their activation in a TLR7/TLR9-dependent fashion [5,15]. Dendritic cells take up these complexes via the Fcγ-receptor IIa (FcγRIIa), transfer them to a subcellular compartment containing FcγRIIa and TLR9 and secrete proinflammatory cytokines and type I interferons [19].

Glucocorticoids, which are used to treat SLE, fail to inhibit TLR-mediated NF-κB activation and fail to reduce type I interferon levels [20]. To improve therapeutic options for SLE and because of the pathophysiological role of nucleic acid-recognizing TLRs in SLE, inhibitory oligonucleotides (INH-ODN) were developed which interfere with the activation of TLR7 (and hTLR8), TLR9 and possibly also with TLR3 to block the stimulatory activity of self-DNA- or self-RNA-complexes. Several classes of INH-ODNs were created among them INH-ODNs with broad and restricted activities on different cell types and were therefore designated B- and R-class INH-ODNs, respectively (reviewed in [21]). B-class INH-ODNs are linear and inhibit several cell types including B-cells, dendritic cells and macrophages while R-class INH-ODNs are palindromic or display short 5’ or 3’ overhangs and inhibit dendritic cells and macrophages but only weakly B-cells [21]. Both classes contain the CC(T)XXX_3–5_GGG motif, which is required for their TLR9-inhibitory activity. Thus, changes within the CCT or GGG sequence reduce while elongation of GGG with additional Gs strengthens the inhibitory potential. Unfortunately, triple and quadruple Gs form complex mixtures of higher order structures or G4-stacks, which make the pharmacological behavior of such INH-ODNs hard to predict and they may cause side effects. We showed recently, that the prototypic INH-ODN 2088 on the one hand blocked TLR7- and 9-mediated responses, but on the other hand, when applied in higher doses, itself triggered murine B-cells TLR9-dependently to proliferate and to secrete IL-6 [22]. Furthermore, this INH-ODN also augmented TLR4-mediated activation of bone marrow-derived macrophages [22]. We therefore developed a series of guanine-modified INH-ODNs where the first or second G nucleotide of the G-quartet was modified to a 7-deaza-2’-deoxyguanosine or a 7-deaza-2’-O-methyl-guanosine. We demonstrated that these INH-ODNs neither formed G4-stacks nor showed the side effects described above and were significantly more potent to inhibit TLR7- and TLR9-induced immune responses in vitro and *in vivo* [22]. Since the inhibitory potential of G-modified INH-ODNs was so far only evaluated using murine immune cells, we now demonstrate their potent activity to impair TLR7- and TLR9-mediated human immune responses.

## Materials and Methods

### Ethic statement

Blood donors (n = 5, age 20–34 years) were healthy volunteers and approved written informed consent about the aims of the study. The local ethic committee of the Klinikum rechts der Isar, Technische Universität München (Munich, Germany) approved the study (project number 37/14). Written consent of volunteers was documented and stored in the secretariat of the Institut für Medizinische Mikrobiologie, Immunologie und Hygiene, Technische Universität München. All animal experiments were reviewed and approved by the local authorities (Regierung von Oberbayern, file number 55.2-1-54-2531-89-10).

### Strains of mice

MRL/Mp-lpr/lpr mice (n = 3, age 12 weeks) were purchased from Harlan Winkelmann GmbH (Borchen, Germany). All mice were kept in the own animal facility under specific pathogen-free conditions. For preparation of immune cells mice were sacrificed by cervical dislocation.

### Reagents

The monoclonal antibodies specific for murine CD45R/B220, CD11b and CD11c were provided by BD Biosciences (Heidelberg, Germany). Human CD20 was purchased from Miltenyi Biotec (Bergisch Gladbach, Germany). Human CD86 was bought from eBioscience (Frankfurt, Germany) and human HLA-DR from Beckman Coulter (Krefeld, Germany). INH-ODNs were provided by Coley Pharmaceutical GmbH (Düsseldorf, Germany) or purchased from BioSpring GmbH (Frankfurt/Main, Germany). Imiquimod was bought from InvivoGen (San Diego, USA), ORN 22075, CpG-ODN 2006 and 2216 were provided by Coley Pharmaceutical GmbH.

### Preparation of immune cells

Murine plasmacytoid BMDCs were generated from the bone marrow of tibiae and femora. Bone marrow cells were plated on bacterial petri-dishes overnight in culture medium (RPMI 1640, 10% heat-inactivated FCS, 100 IU/ml penicillin, 100 μg/ml streptomycin (PAA Laboratories GmbH, Pasching, Austria) and 50 μM 2-ME (Invitrogen, Carlsbad, USA)) to remove adherent cells. Non-adherent cells were directly plated on 6 well plates at a density of 4.5x10^6^ cells/well and cultivated for 7–8 days in complete medium in the presence of FLT3 ligand (R&D Systems Europe, Ltd., Abingdon, United Kingdom) to mature the cells. The medium was additionally supplemented with sodium pyruvate 1%, NEA 1%, L-Glutamine 1% (PAA Laboratories GmbH, Pasching, Austria). FACS analysis demonstrated that the majority of cells obtained were CD45R/B220 high and CD11b low.

Human peripheral blood mononuclear cells (PBMCs) were prepared from blood of volunteers by Ficoll (Biochrom AG, Berlin, Germany) density gradient centrifugation. Human B-cells were positively selected via magnetic cell separation using anti-CD19 microbeads (Miltenyi Biotec). FACS analysis demonstrated that 95% of the cells obtained were CD20^+^ B-cells.

### Inhibition assay

Murine pDCs, human PBMCs or B-cells were stimulated with the TLR9 agonists CpG-ODN 2006 or 2216 or TLR7 agonists ORN 22075 (R-1075, C*C*G*U*C*U*G*U*U*G*U*G*U*G*A*C*U*C) [23], imiquimod or R848 in the presence of 10-fold titrated amounts of INH-ODNs (0.01–10 μM). The medium used was RPMI supplemented with 10% FCS, 100 IU/ml penicillin, 100 μg/ml streptomycin (PAA Laboratories GmbH, Pasching, Austria) and in case of murine cells 50 μM 2-ME. Cytokine levels in the supernatant were determined after 24 hours to 6 days of culture in 96 well microtiterplates (Falcon, Colorado, USA).

Determination of cytokines, intracellular ATP- and extracellular LDH-levels Human IL-6 (R&D Systems Europe, Ltd., United Kingdom) and human IFN-α (eBioscience, Frankfurt, Germany) were determined using commercially available ELISA kits. Murine IFN-α was measured using antibodies from tebu-bio GmbH (Offenbach, Germany) and Jackson Immuno Research Europe ltd. (Suffolk, United Kingdom). The assays were performed according to the manufacturer’s manual. Intracellular ATP-levels were measured by CellTiter-Glo Luminescent Cell Viability Assay (Promega, Madison, USA). Briefly, cells were washed and subsequently lysed with CellTiter-Glo Buffer. The ATP-content of the lysate was measured via luminometer (Berthold Titertek Instruments, Pforzheim, Germany). Extracellular LDH-levels were determined using CytoTox 96 Non-Radioactive Cytotoxicity Assay. Data were analyzed using SigmaPlot 12.0 (Systat Software, USA).

### Flow cytometry

PBMCs were stained with CD20, CD86 and HLA-DR antibodies. CD20^+^ cells were assayed for CD86 and HLA-DR expression. Flow cytometry was performed with a FACS Calibur instrument (BD Biosciences, San Jose, CA, USA) or a CyAn ADP9 color device (Beckman Coulter, Krefeld, Germany), the data were analysed using the FlowJo software (Tree Star Inc, OR, USA).

### Statistics

More than two equally treated groups were tested for significant differences with one way ANOVA, post hoc test Holm-Sidak. Statistical analysis was performed with SigmaPlot (Systat Software, USA).

## Results

### Basic characteristics of INH-ODNs

Basic properties of our series of INH-ODNs used in this study are listed in Table 1 and were reported previously [22]. Briefly, INH-ODN 2088 contained the TLR9-inhibition motif CC(T)XXX_3–5_GGG, was characterized as a TLR3, 7 and 9 inhibitor in the murine system and formed G-tetrads. It efficiently impaired IFN-α release by murine plasmacytoid dendritic cells (pDCs) but had limited inhibitory activity on murine bone marrow-induced macrophages (BMDM) and murine B-cells and revealed unexpected side effects [22]. By G-modification of INH-ODN 2088 we generated INH-ODNs 21595, 20844 and 24888 (Table 1). All of them were equally or significantly more potent than INH-ODN 2088 to impair TLR7- or TLR9-induced responses by murine immune cells. INH-ODN 24888 also impaired TLR3-mediated induction of IL-12p40, which was not evaluated for the other two INH-ODNs. The sequence of INH-ODN 21158 consisted only of the TLR9-inhibition motif CC(T)XXX_3–5_GGG (Table 1). As expected this INH-ODN impaired TLR9- but influenced TLR7-driven immune responses only weakly [22]. Furthermore, it reduced TLR3-mediated IL-12p40 secretion by BMDMs. Both G-modified derivatives of INH 21158, INH-ODN 24987 and 24991, were significantly more efficient to impair TLR9, the latter unexpectedly also impeded TLR7- and TLR3-mediated immune responses. INH-ODN 20959 and the G-modified variants INH-ODNs 105870 and 105871 did not contain a TLR9-inhibition motif and the latter two preferentially impaired TLR7 (Table 1). INH-ODN 105871 also impeded TLR3; this ability was not evaluated for INH-ODN 105870 [22]. All G-modified INH-ODNs neither activated murine B-cells nor enhanced significantly cellular responses induced via TLR2 or TLR4 [22].

**Table 1:** Properties of INH-ODNs.

INH-ODN	sequence	reported TLR-inhibitory activity [22]	reduced G-tetrade formation
**2088**	T*C*C*T*G*G*C*G*G*G*G*A*A*G*T	3, 7, 9	no
21595	T*C*C*T*G*G*C*E*G*G*G*A*A*G*T	7, 9	yes
20844	T*C*C*T*G*G*C*G*E*G*G*A*A*G*T	7, 9	yes
24888	T*C*C*T*G*G*C*mE*G*G*G*A*A*G*T	3, 7, 9	yes
**21158**	C*C*T*G*G*C*G*G*G*G	3, 9	no
24987	C*C*T*G*G*C*E*G*G*G	9	yes
24991	C*C*T*G*G*C*mE*G*G*G	3, 7, 9	yes
**20959**	T*A*A*T*G*G*C*G*G*G*G*A*A*G*T	n.d.	n.d.
105870	T*A*A*T*G*G*C*E*G*G*G*A*A*G*T	7	yes
105871	T*A*A*T*G*G*C*mE*G*G*G*A*A*G*T	3, 7	yes

Bold INH-ODN designations indicate unmodified parent INH-ODNs which define the sequence for the following G-modified variants. Underlined residues indicate TLR9-inhibition motif. * = phosphorothioate-binding, E = 7-deaza-2’-deoxyguanosine, mE = 7-deaza-2’-O-methyl-guanosine. G-tetrade formation of INH-ODN was analyzed by SEC-HPLC. n.d. not determined

### Stimulatory potential of TLR7- or TLR9-ligands for human PBMCs

**Fig 1:**
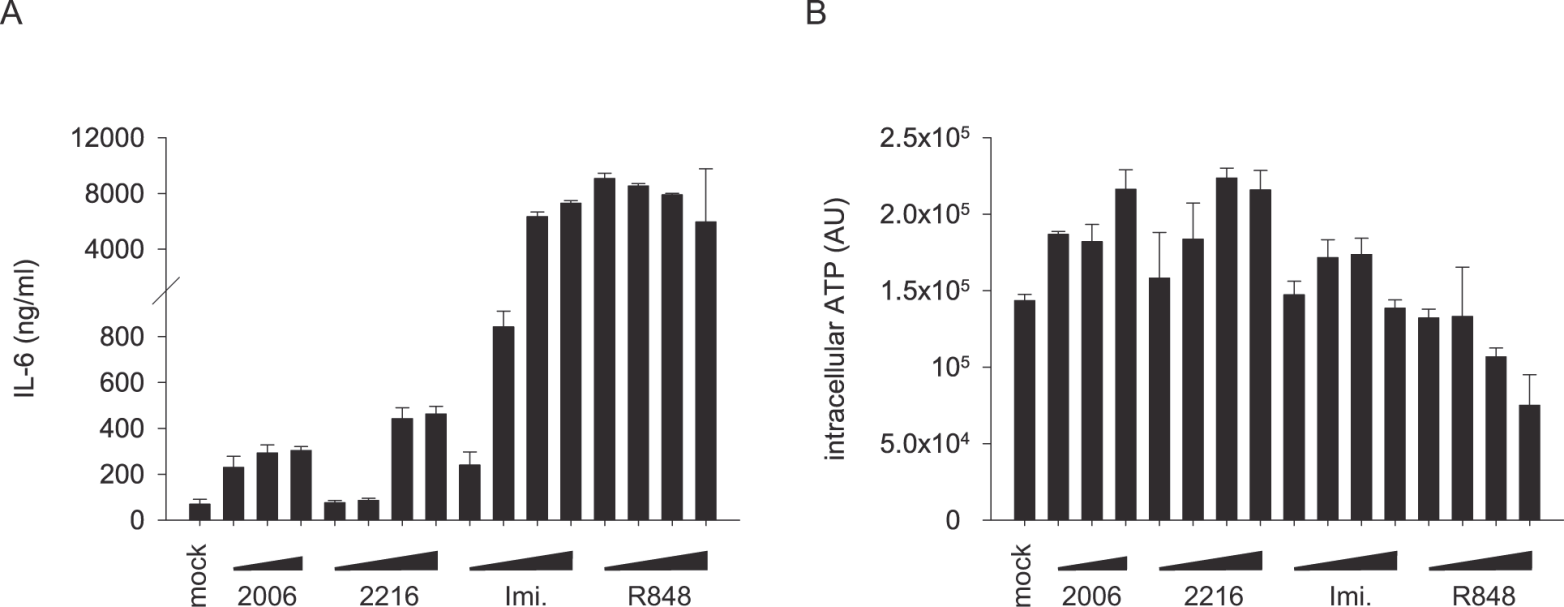
Dose response relationship of TLR7- and TLR9-ligands. (A) Human PBMCs (2x10^5^ cells/well) were stimulated with the TLR9-ligands CpG-ODN 2006 (1, 5, 10 μM) or CpG-ODN 2216 (0.5, 1, 5, 10 μM) or with the TLR7-ligands imiquimod (0.5, 1, 5, 10 μM) or R848 (0.5, 1, 5, 10 μM). After a culture period of 4 days IL-6 was quantified in the culture supernatant by ELISA. (B) The intracellular ATP content of the cells stimulated in (A) was analyzed. Error bars represent SD of three individual cultures from one donor.

Before we examined the inhibitory potential of G-modified INH-ODNs for human immune cells, we evaluated the stimulatory activity of TLR7- and TLR9-ligands, since their activity counteracts the inhibitory potency of INH-ODNs. PBMCs were induced to secrete IL-6 by the two TLR7-ligands imiquimod and R848 as well as the TLR9-ligands CpG-ODN 2006 and 2216. CpG-ODN 2006 was chosen as a powerful stimulator of human B-cells [24] while CpG-ODN 2216 is a known stimulator of type I interferons by pDCs [25]. Interestingly, we found that the dose-response relationship of TLR9- versus TLR7-agonists to induce IL-6 secretion by PBMCs differed. Thus, IL-6 levels were much lower upon stimulation with both CpG-ODNs than with imiquimod or R848 (Fig. 1A). In addition, we evaluated the intracellular ATP-content of PBMCs as a parameter for cellular proliferation but also cytotoxicity [26]. We found that both TLR9-stimuli dose-dependently increased the intracellular ATP-content, while imiquimod did not influence this parameter and high doses of R848 lowered the amount of ATP which may indicate some degree of cytotoxicity (Fig. 1B). Taken together it appeared that TLR9-ligands were inferior to TLR7-agonists to induce IL-6 secretion by human PBMCs but superior to increase intracellular ATP-levels.

### G-modified INH-ODNs were not toxic for human PBMCs

**Fig 2:**
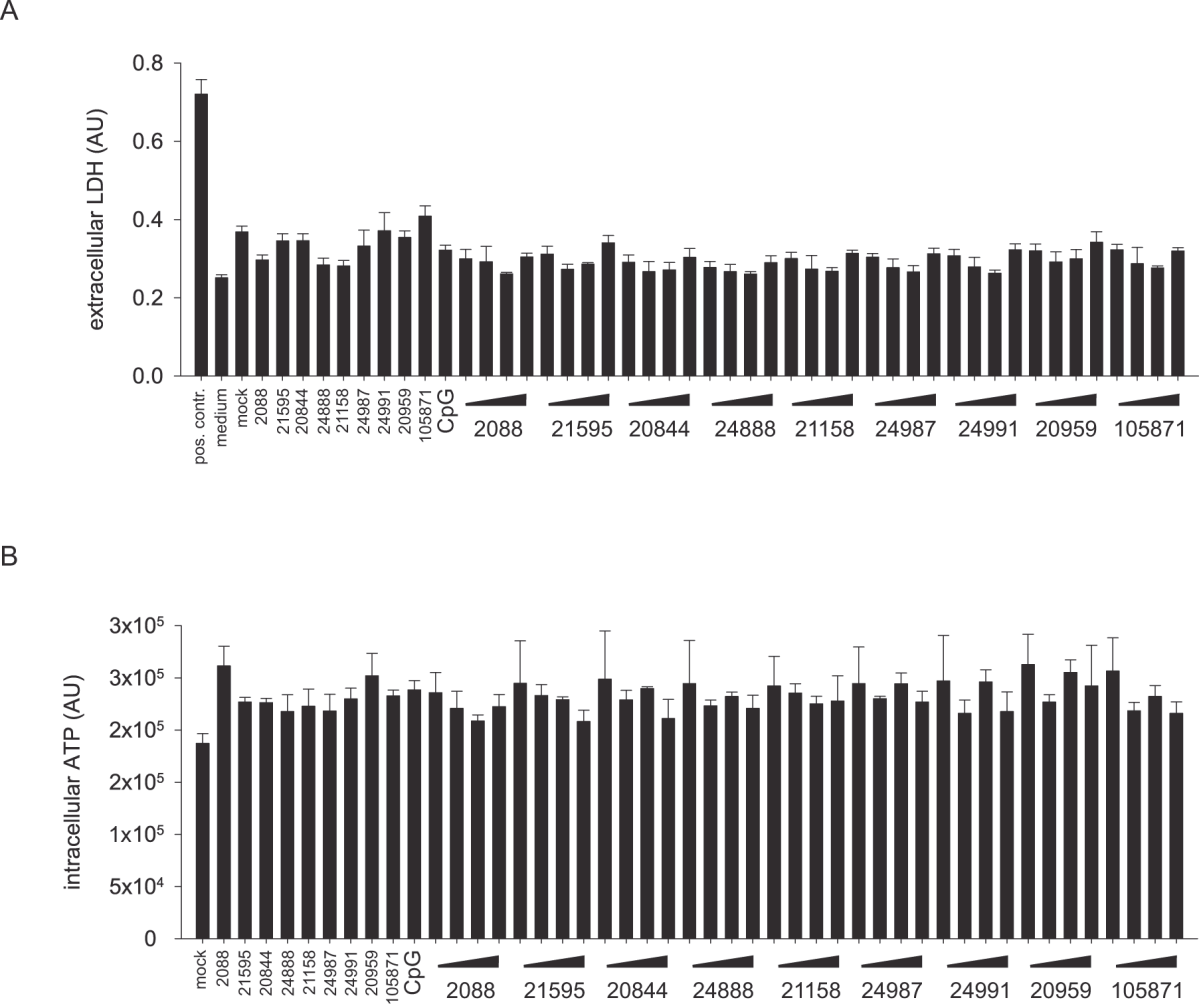
INH-ODNs are not toxic for human PBMCs. (A) PBMCs (4x10^5^ cells/well) were cultured with INH-ODNs alone (10 μM) or with a combination of CpG-DNA 2006 (100 nM) and titrated amounts of INH-ODNs (0.01, 0.1, 1, 10 μM). Medium in the absence of cells (medium) and cells cultured in the absence of CpG-DNA 2006 or INH-ODNs (mock) served as negative controls. Bovine LDH was used as positive control (pos. contr.). The extracellular LDH-content was determined after 48h of culture. Error bars represent SD of three individual cultures. The experiment was repeated with imiquimod (5 μg/ml) using cells from a different donor (data not shown). INH-ODNs were again not toxic. (B) PBMCs (4x10^5^ cells/well) were not stimulated (mock) or stimulated with CpG-ODN 2006 (100 nM) or a combination of CpG-ODN 2006 (100 nM) and titrated amounts of INH-ODNs (0.01, 0.1, 1, 10 μM) as indicated. The intracellular ATP-content was determined after 48h of culture. Error bars represent SD of three individual cultures. The experiment was repeated twice with cells from another donor with similar results.

The cytotoxic potential of INH-ODNs for PBMCs was evaluated by quantification of the extracellular release of the cytosolic protein lactate dehydrogenase (LDH). Incubation of PBMCs with INH-ODNs alone or in combination with the stimulatory CpG-ODN 2006 (or imiquimod, data not shown) did not result in increased levels of extracellular LDH (Fig. 2A). Moreover, intracellular ATP-levels of CpG-ODN 2006-stimulated PBMCs were not reduced below the level of mock-treated cells by INH-ODNs (Fig. 2B). Taken together, we found no evidence that the series of INH-ODNs used in this study was toxic for PBMCs.

### G-modified INH-ODNs prevent IFN-α secretion by PBMCs

**Fig 3:**
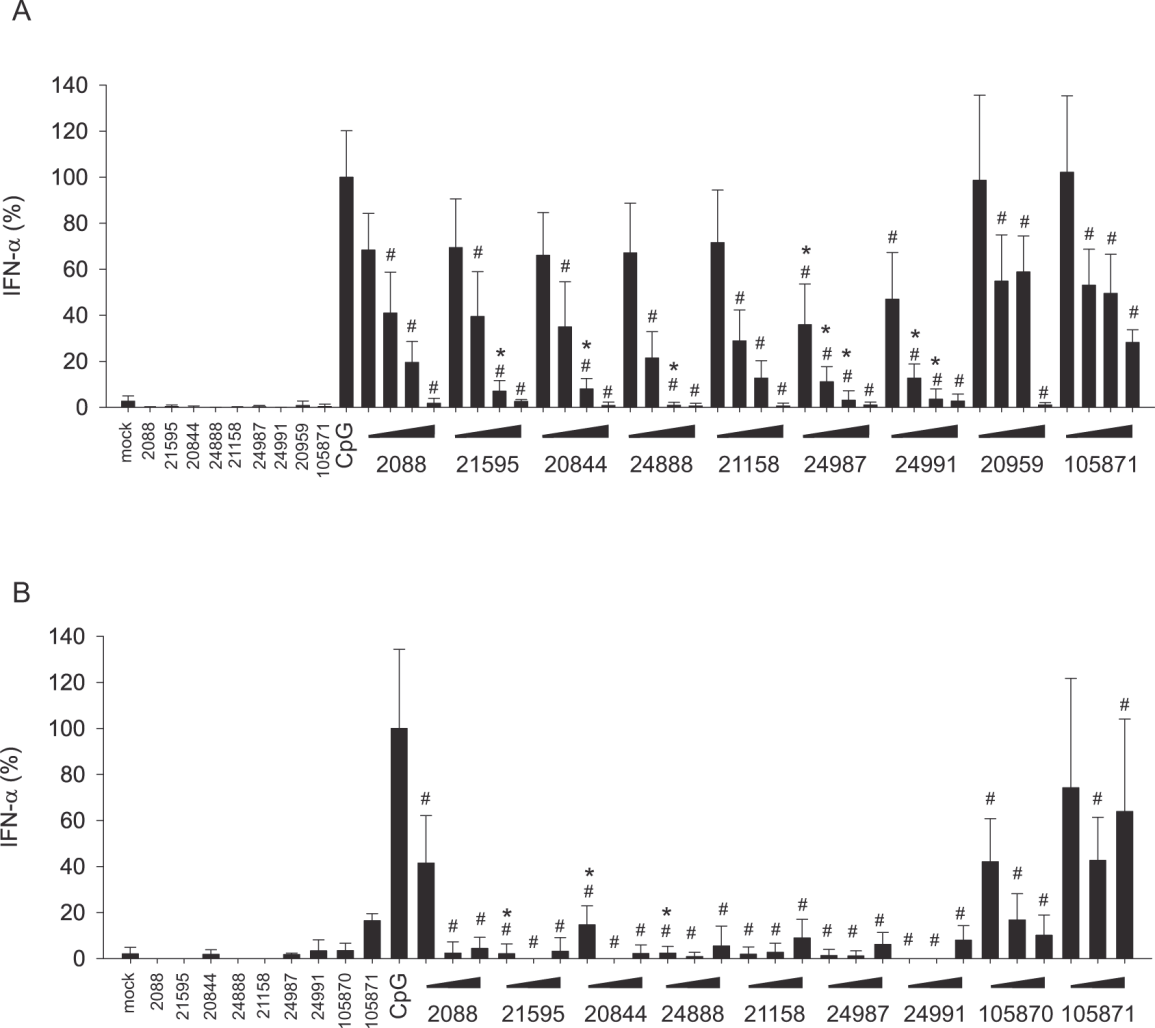
INH-ODNs impair efficiently TLR9-mediated release of IFN-α by PBMCs. (A) Human PBMCs (5–6x10^5^ cells/well) were stimulated with CpG-ODN 2216 (3 μM) in the absence (CpG) or presence of titrated amounts of INH-ODNs (0.01, 0.1, 1, 10 μM) for 24h. IFN-α was determined in the culture supernatant by ELISA. To determine whether INH-ODNs influence IFN-α release per se, the highest dose (10 μM) of each INH-ODN was also evaluated without TLR-mediated stimulation. Data represent mean and SD of three independent experiments, each experiment was performed with cells from a different donor (each bar represents n = 6–9 cultures). ^#^p<0.05, ANOVA compared to CpG-ODN 2216; *p<0.05, ANOVA compared to INH-ODN 2088 for G-modified INH-ODNs 21595, 20844 and 24888 and compared to INH-ODN 21158 for G-modified INH-ODN 24987 and 24991. (B) For comparison bone marrow-derived pDCs (2x10^5^ cells/well) from lupus-prone MRL/Mp-lpr/lpr mice were treated as described in (A) with the exception that instead of INH-ODN 20959 G-modified INH-ODN 105870 was used. INH-ODN were used at a concentration of 0.1, 1, 10 μM. Data represent mean and SD of two separate experiments (each bar represents n = 2–6 cultures). ^#^p<0.05, ANOVA compared to CpG-ODN 2216; *p<0.05, ANOVA compared to INH-ODN 2088 for G-modified INH-ODNs 21595, 20844 and 24888.

**Fig 4:**
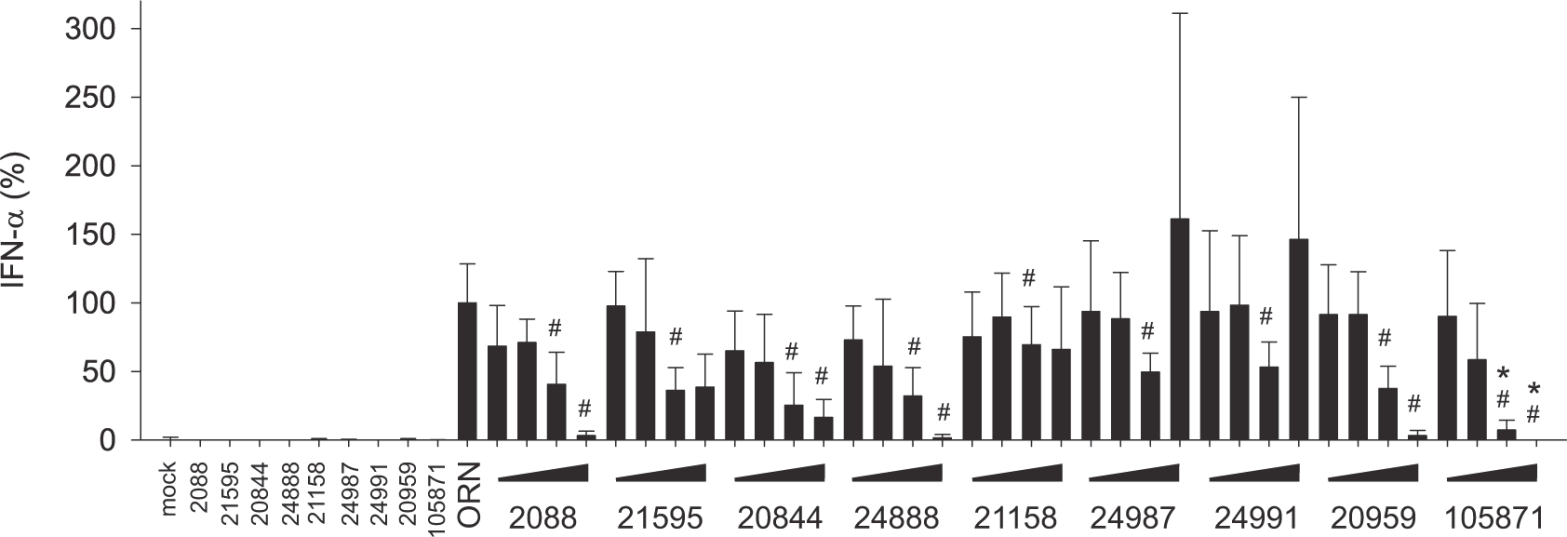
IFN-α release triggered via TLR7 is also efficiently impaired by INH-ODNs. The experiment was performed as described in Fig. 3A with the exception that PBMCs were stimulated with the TLR7-ligand RNA-ORN 22075 (5 μM). Data represent mean and SD of three independent experiments, each experiment was performed with cells from a different donor (each bar represents n = 9 cultures). ^#^p<0.05, ANOVA compared to RNA-ORN; *p<0.05, ANOVA compared to INH-ODN 20959 for G-modified INH-ODN 105871.

Type I interferons are considered as crucial to maintain inflammation in SLE [4]. Thus, we evaluated whether G-modified INH-ODNs would impair the secretion of these cytokines by PBMCs stimulated either with the TLR-ligand CpG-ODN 2216 or the TLR7-ligand RNA-ORN 22075. We used the TLR7 agonist RNA-ORN 22075 instead of imiquimod in this experimental setting since the former induces a considerably stronger IFN-α response. All G-modified INH-ODNs containing the TLR9-inhibition motif were significantly more effective to inhibit the release of IFN-α as the parent INH-ODNs 2088 or 21158 while INH-ODNs 20959 and 105871, which lack such a motif, displayed a much weaker inhibitory activity as expected (Fig. 3A). Virtually the same inhibition pattern was observed with murine pDC from autoimmune MRL/MP-lpr/lpr mice stimulated with the CpG-ODN 2216 (Fig. 3B). Since pDCs are the main producers of type I interferons within human PBMCs [27] we assume that INH-ODNs also inihibited human pDCs, although we did not directly analyze this cell type. The secretion of IFN-α by PBMCs stimulated via TLR7 was also efficiently impaired by G-modified INH-ODNs, with the exception of INH-ODNs whose sequence consisted only of a TLR9-inhibition motif (Fig. 4). These INH-ODNs influenced secretion of IFN-α only weakly as expected.

### G-modified INH-ODNs are more efficient to inhibit IL-6 secretion of PBMCs

**Fig 5:**
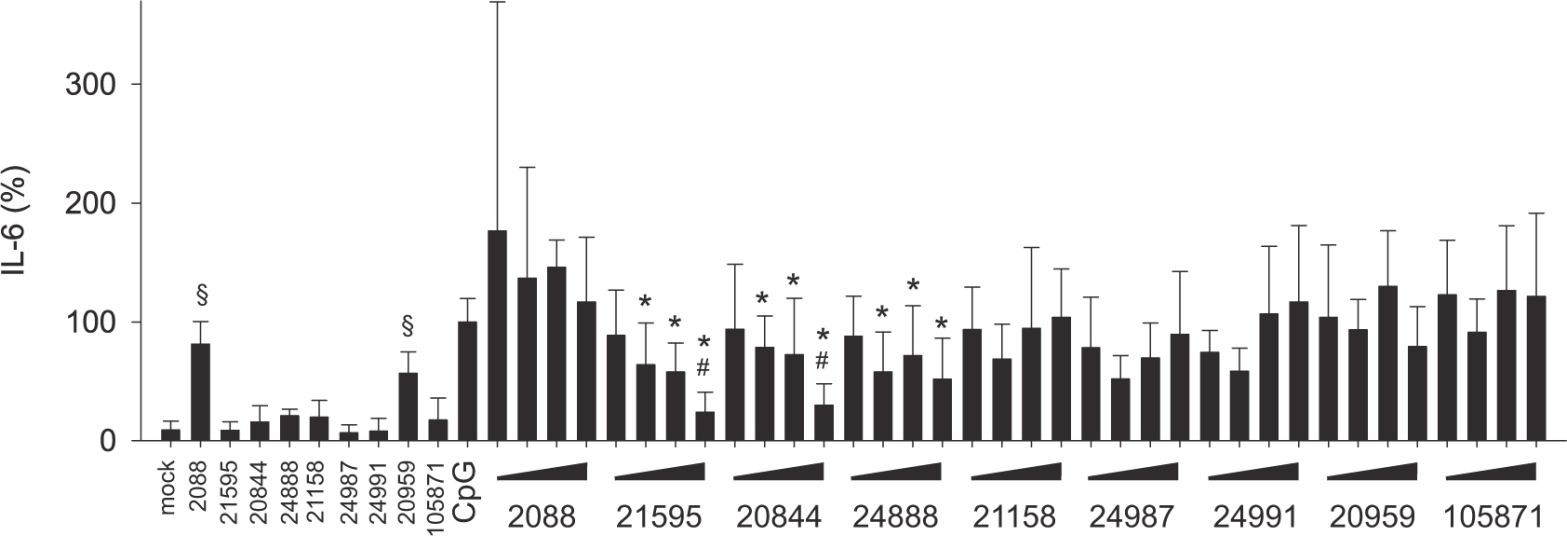
G-modified INH-ODNs were significantly more effective in preventing IL-6 release by CpG-ODN-stimulated human PBMCs. PBMCs (3–4x10^5^ cells/well) were stimulated with CpG-ODN 2006 (100 nM) in the absence (CpG) or presence of titrated amounts of INH-ODNs (0.01, 0.1, 1, 10 μM) for 48h. IL-6 was determined in the culture supernatant by ELISA. To determine whether INH-ODNs influence IL-6 release per se, the highest dose (10 μM) of each INH-ODN was also evaluated without TLR-mediated stimulation. Data represent mean and SD of three independent experiments, each experiment was performed with cells from a different donor (each bar represents n = 8–9 cultures). ^#^p<0.05, ANOVA compared to CpG-ODN 2006; *p<0.05, ANOVA compared to INH-ODN 2088 for G-modified INH-ODNs 21595, 20844 and 24888; ^§^p<0.05, ANOVA compared to mock in the absence of a TLR-stimulus.

**Fig 6:**
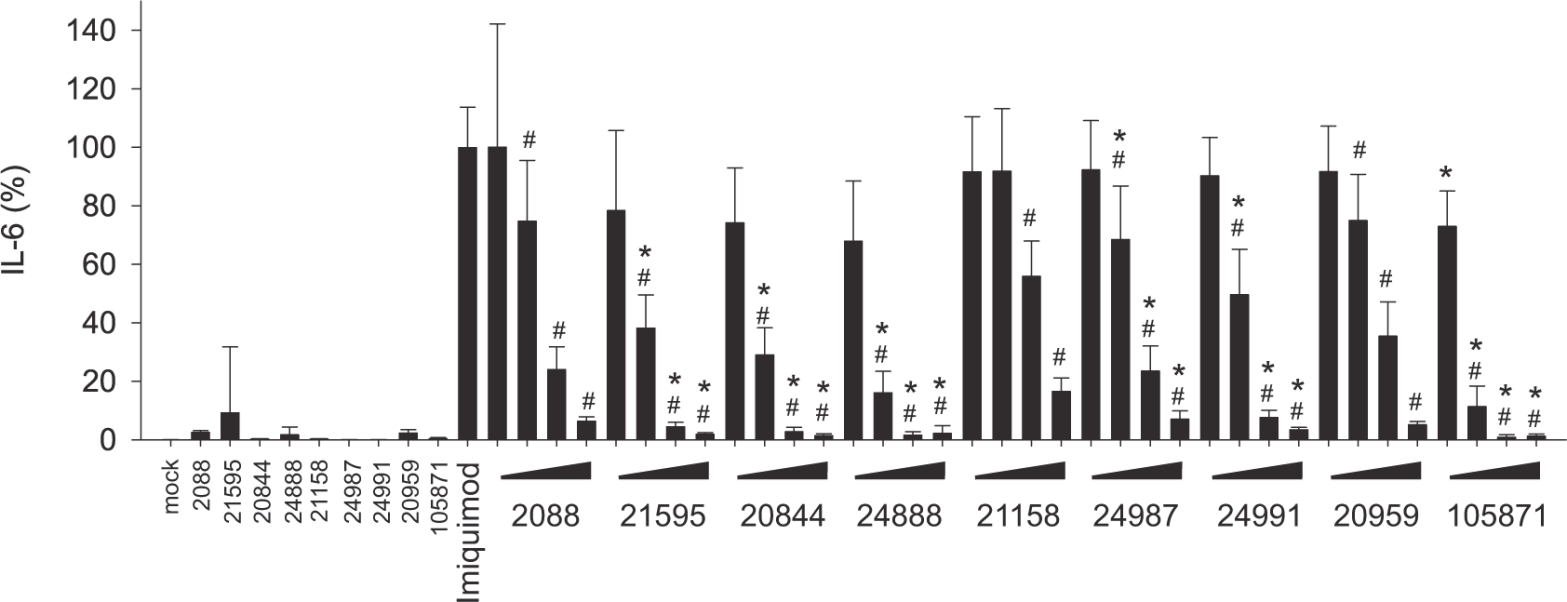
G-modification significantly improves the ability of INH-ODNs to impair imiquimod-induced IL-6 release by human PBMCs. PBMCs (4x10^5^ cells/well) were stimulated with imiquimod (5 μg/ml) in the absence (imiquimod) or presence of titrated amounts of INH-ODNs (0.01, 0.1, 1, 10 μM) for 48h. IL-6 was determined in the culture supernatant by ELISA. To determine whether INH-ODNs influence IL-6 release per se, the highest dose (10 μM) of each INH-ODN was also evaluated without TLR-mediated stimulation. Data represent mean and SD of two independent experiments, each experiment was performed with cells from a different donor (each bar represents n = 6 cultures). ^#^p<0.05, ANOVA compared to imiquimod; *p<0.05, ANOVA compared to INH-ODN 2088 for G-modified INH-ODNs 21595, 20844 and 24888 or compared to INH-ODN 21158 for G-modified INH-ODN 24987 and 24991 or compared to INH-ODN 20959 in case of G-modified INH-ODN 105871.

As reported earlier, high doses of INH-ODN 2088 unexpectedly stimulated IL-6-secretion of murine B-cells and thereby compromised its inhibitory activity [22]. Therefore, we incubated human PBMCs with our series of INH-ODNs in the absence of any further TLR-ligand and measured the release of IL-6. We observed that two out of three non-G-modified INH-ODNs, namely INH-ODN 2088 and 20959, stimulated PBMCs to release IL-6 (Fig. 5). G-modification of INH-ODNs largely abolished this activity (Fig. 5). INH-ODN 2088 and 21158 failed to inhibit IL-6 release of PBMCs stimulated with CpG-ODN 2006, while the G-modified INH-ODNs 21595, 20844 and 24888 impaired this response (Fig. 5). Modified INH-ODNs 24987 and 24991 were much weaker inhibitors. As expected, both INH-ODNs lacking the TLR9-inhibition motif, INH-ODN 20959 and 105871 did not influence IL-6 release (Fig. 5). PBMCs stimulated with the TLR7-ligand imiquimod again produced much higher levels of IL-6 compared to stimulation with the TLR9-ligand CpG-ODN 2006 (data not shown). All G-modified INH-ODNs blocked IL-6 release significantly better compared to their unmodified precursors (Fig. 6). Although INH-ODNs 21158, 24987, 24991, whose sequence consisted only of the TLR9-inhibition motif, did not influence TLR7-driven release of IFN-α (Fig. 4), they surprisingly impaired TLR7-mediated IL-6-release by human PBMCs. Taken together, G-modification of INH-ODNs largely reduced their self-stimulatory activity, which can be observed across species, i.e. with human and murine immune cells. This explains in part that their inhibitory efficacy is significantly improved.

### Inhibition of human B-cells by G-modified INH-ODNs

**Fig 7:**
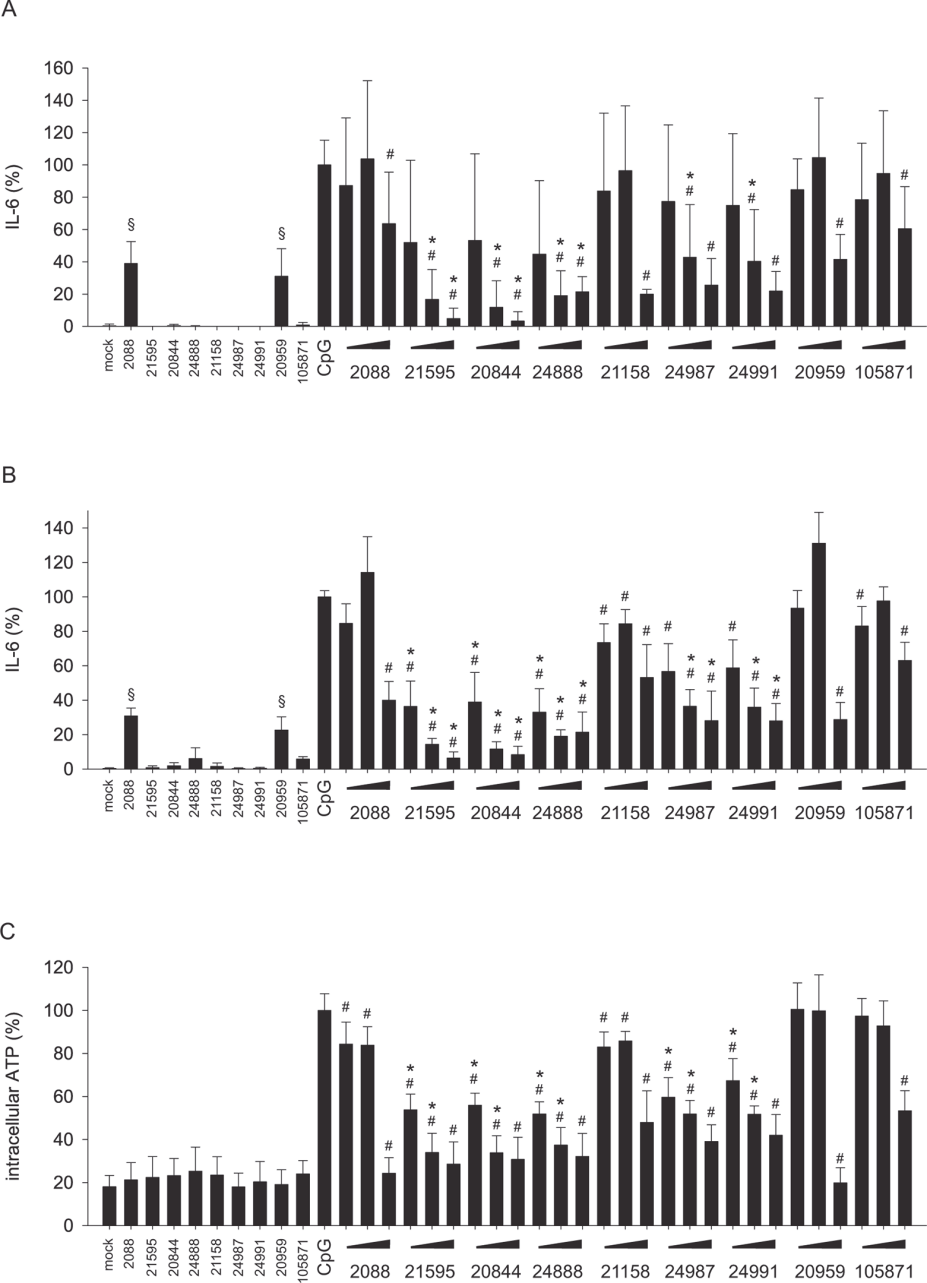
IL-6 release by CpG-ODN-activated human B-cells is significantly more prevented by G-modified INH-ODNs. (A) B-cells (5–10x10^4^ cells/well) were stimulated with CpG-ODN 2006 (100 nM) in the absence (CpG) or presence of titrated amounts of INH-ODNs (0.1, 1, 10 μM) for 24h. IL-6 was determined in the culture supernatant by ELISA. To determine whether INH-ODNs influence IL-6 release per se, the highest dose (10 μM) of each INH-ODN was also evaluated without TLR-mediated stimulation. Data represent mean and SD of three independent experiments, each experiment was performed with cells from a different donor (each bar represents n = 4–9 cultures). (B) IL-6 content of the culture supernatants of the experiment described in (A) was additionally determined after 5 days of culture. (C) depicts the intracellular ATP-content of the cells described in (B). Data represent mean and SD of three independent experiments, each experiment was performed with cells from a different donor (each bar represents n = 4–9 cultures). ^#^p<0.05, ANOVA compared to CpG-ODN 2006; *p<0.05, ANOVA compared to INH-ODN 2088 for G-modified INH-ODNs 21595, 20844 and 24888 or compared to INH-ODN 21158 for G-modified INH-ODN 24987 and 24991; ^§^p<0.05, ANOVA compared to mock in the absence of a TLR-stimulus.

**Fig 8:**
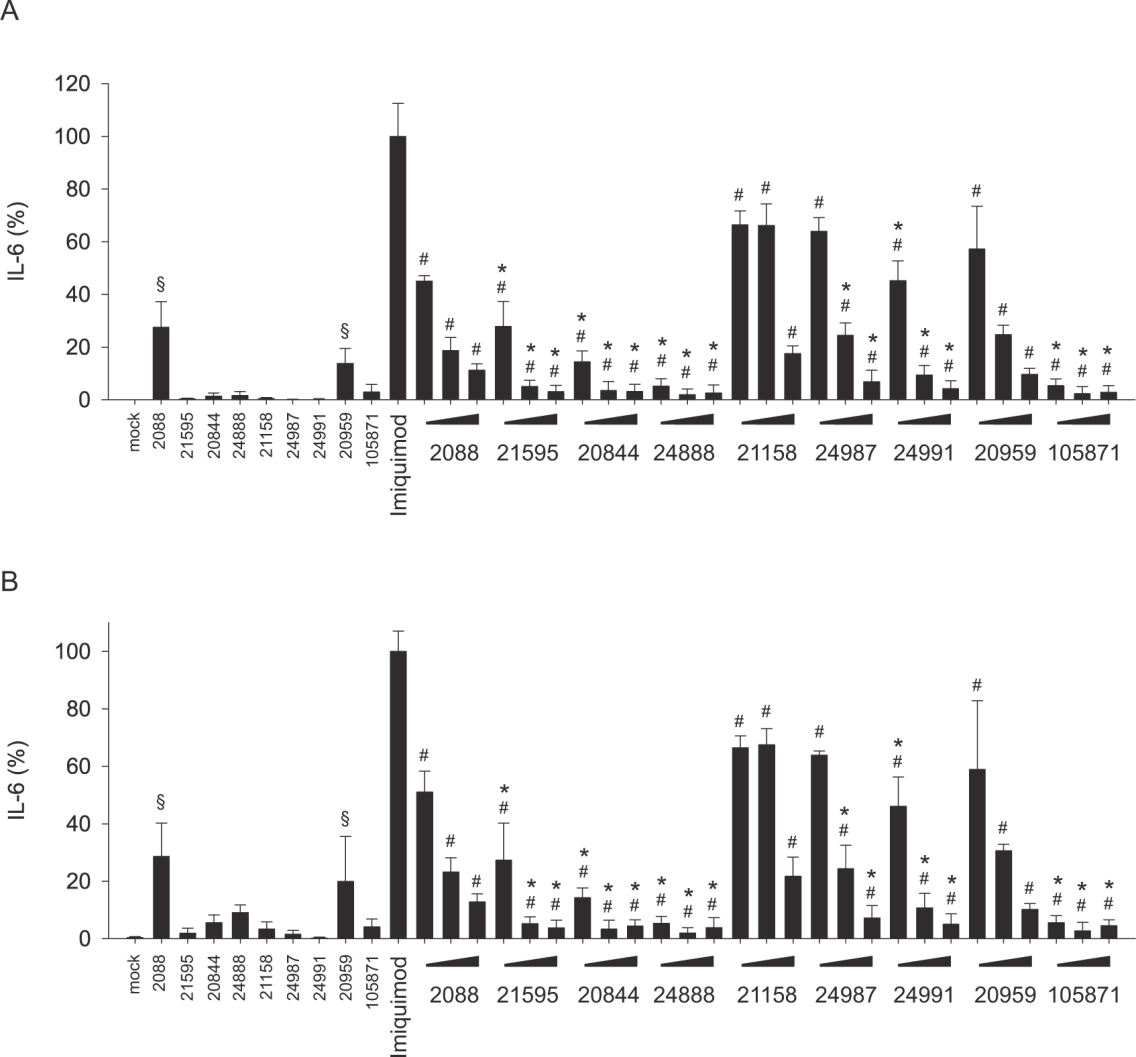
G-modified INH-ODNs are significantly more effective to inhibit IL-6 release by imiquimod-stimulated human B-cells. (A) B-cells (1x10^5^ cells/well) were stimulated with imiquimod (5 μg/ml) in the absence (imiquimod) or presence of titrated amounts of INH-ODNs (0.1, 1, 10 μM) for 24h. IL-6 was determined in the culture supernatant by ELISA. To determine whether INH-ODNs influence IL-6 release per se, the highest dose (10 μM) of each INH-ODN was also evaluated without TLR-mediated stimulation. Data represent mean and SD from two independent experiments, each experiment was performed with cells from a different donor (each bar represents n = 3–6 cultures). (B) IL-6 content of the culture supernatants of the experiment described in (A) was additionally determined after 6 days of culture. ^#^p<0.05, ANOVA compared to imiquimod; *p<0.05, ANOVA compared to INH-ODN 2088 for G-modified INH-ODNs 21595, 20844 and 24888 or compared to INH-ODN 21158 for G-modified INH-ODN 24987 and 24991 or compared to INH-ODN 20959 in case of G-modified INH-ODN 105871; ^§^p<0.05, ANOVA compared to mock in the absence of a TLR-stimulus.

Since only human pDCs and B-cells express high levels of TLR7 and TLR9 [28] and both cell types are involved in the pathophysiology of SLE, we also explored the inhibitory potential of G-modified INH-ODNs on human B-cells stimulated via TLR7 or TLR9. Incubation of human B-cells with INH-ODNs alone again revealed the self-stimulatory activity of INH-ODN 2088 and 20959 (Fig. 7A, B) as already described for human PBMCs (Fig. 5). G-modified INH-ODNs were not stimulatory. INH-ODN 2088 hardly inhibited IL-6 release by B-cells stimulated with CpG-DNA 2006 (Fig. 7A). All G-modified derivatives of INH-ODN 2088 efficiently impaired IL-6 release and were more effective than INH-ODN 21158 and its G-modified variants INH-ODN 24987 and 24991 (Fig. 7A). INH-ODNs 20959 and 105871, which lack the TLR9 inhibition motif, were least effective as expected (Fig. 7A). Extension of the incubation period from 24h to 5d did not change the observed inhibition pattern of INH-ODNs (compare Fig. 7A and B) and thus indicated that prolonged stimulation of the cells with CpG-DNA, which was used in its stabile phosphorothioate form, could not override inhibition by G-modified INH-ODNs (Fig. 7B). The inhibition pattern of INH-ODNs was also not altered when an alternative parameter of cellular activation, the intracellular ATP-content, was analyzed (Fig. 7C). In contrast to IL-6 release, unmodified INH-ODNs did not increase this parameter in the absence of CpG-ODN 2006 (Fig. 7C). Imiquimod-induced IL-6 secretion of human B-cells was inhibited by INH-ODN 2088 to some extent, but the G-modified derivatives INH-ODN 21595, 20844 and 24888 were significantly more efficient (Fig. 8A). Unexpectedly, INH-ODN 21158 and more efficiently G-modified INH-ODNs 24987 and 24991 also impaired this B-cell response. Comparison of INH-ODN 20959 and its G-modified variant INH-ODN 105871 again revealed that G-modification significantly increased the inhibitory efficacy. Prolonged stimulation of B-cells with imiquimod could also not override inhibition by INH-ODNs. Again, unmodified INH-ODNs stimulated IL-6 secretion by themselves (Fig. 8A, B).

**Fig 9:**
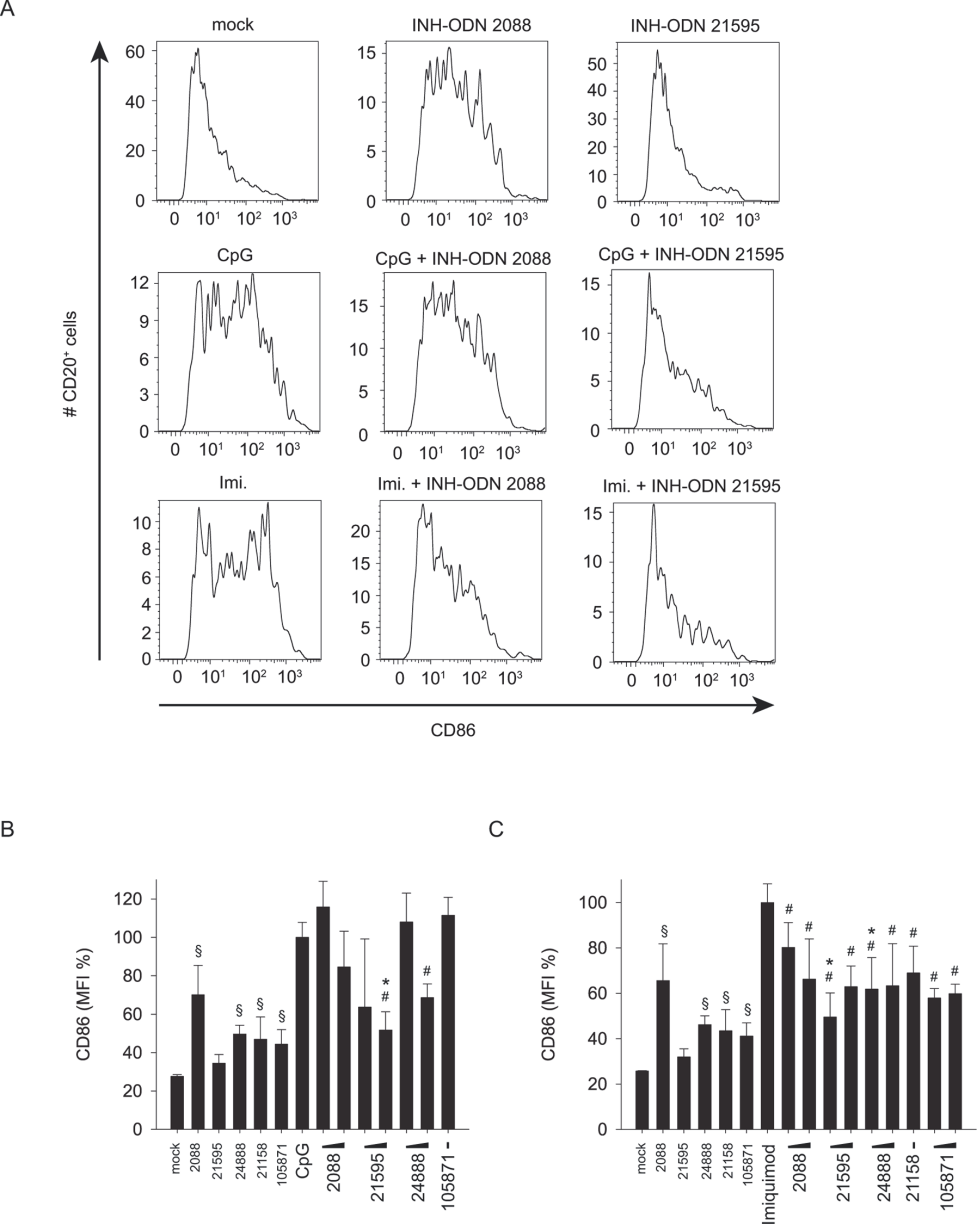
G-modified INH-ODNs prevent CpG-ODN or imiquimod induced CD86 expression by CD20^+^ B-cells. PBMCs (4x10^5^ cells/well) were stimulated with CpG-ODN 2006 (100 nM) or imiquimod (5 μg/ml) in the absence or presence of INH-ODNs (1, 10 μM). After 48h cells were harvested, stained with CD20 and CD86 antibodies and CD20^+^ cells were analyzed by flow cytometry for CD86 expression. (A) shows CD86 expression profiles upon treatment with individual stimulator/inhibitor combinations as indicated. (B) and (C) depict CD86 expression post stimulation with CpG-ODN 2006 (B) or imiquimod (C) from two independent experiments. Data represent mean and SD of these two experiments, each experiment was performed with cells from a different donor (each bar represents n = 4 cultures). ^#^p<0.05, ANOVA compared to CpG-ODN 2006; *p<0.05, ANOVA compared to INH-ODN 2088 for G-modified INH-ODNs 21595 and 24888 ^§^p<0.05, ANOVA compared to mock in the absence of a TLR-stimulus.

**Fig 10:**
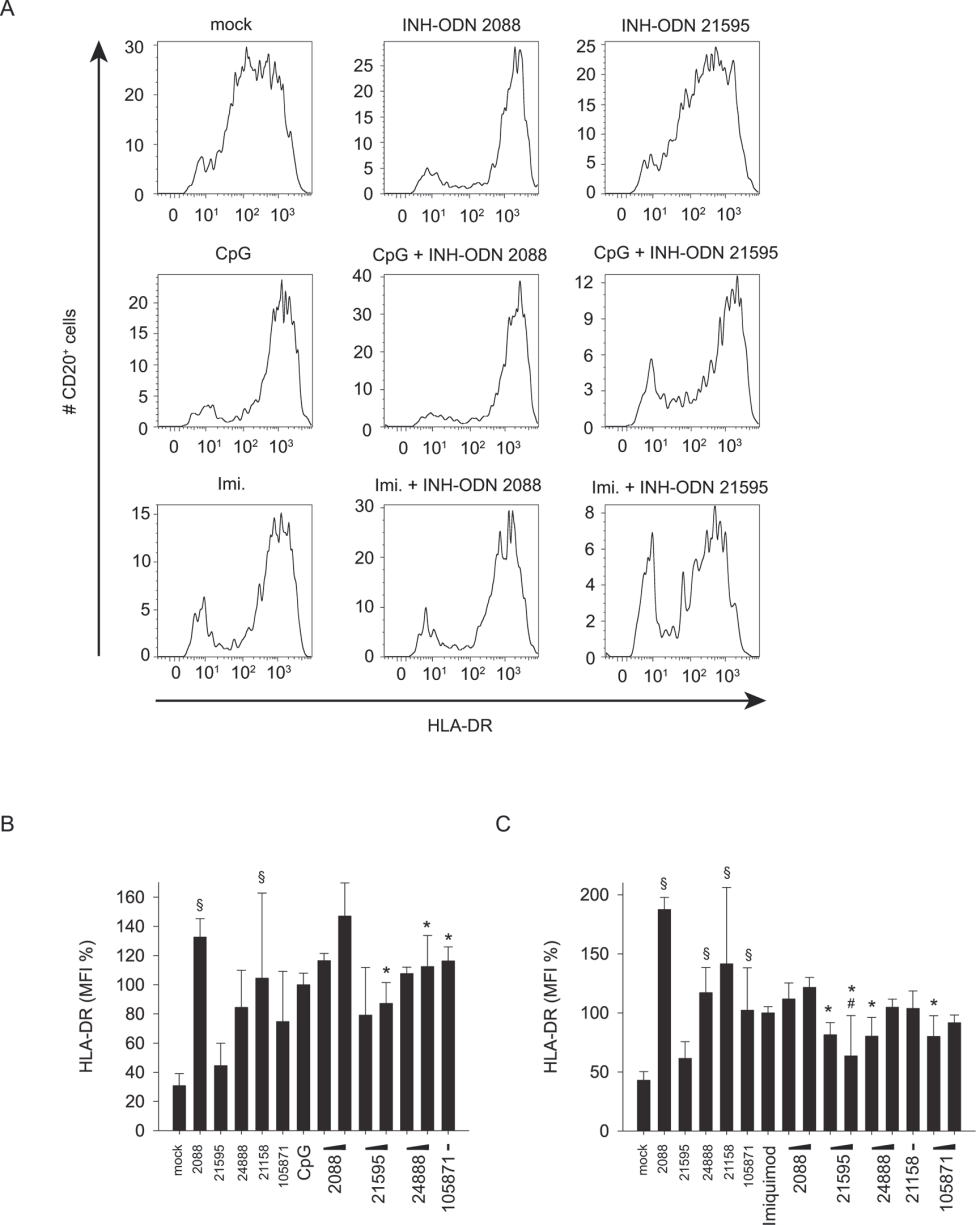
G-modified INH-ODNs partially prevent CpG-ODN or imiquimod induced HLA-DR expression by CD20^+^ B-cells. The cells used in Fig. 9 were also stained with an HLA-DR antibody and analyzed by flow cytometry for HLA-DR expression. (A) shows HLA-DR expression profiles upon treatment with individual stimulator/inhibitor combinations as indicated. (B) and (C) depict HLA-DR expression post stimulation with CpG-ODN 2006 (B) or imiquimod (C) from two independent experiments. Data represent mean and SD of these two experiments, each experiment was performed with cells from a different donor (each bar represents n = 4 cultures). ^#^p<0.05, ANOVA compared to CpG-ODN 2006; *p<0.05, ANOVA compared to INH-ODN 2088 for G-modified INH-ODNs 21595 and 24888 ^§^p<0.05, ANOVA compared to mock in the absence of a TLR-stimulus.

Stimulation of human B-cells with CpG-ODN 2006 or imiquimod increased the expression of CD86 and HLA-DR (Fig. 9, 10). We therefore analyzed whether G-modified INH-ODNs were able to prevent the induced expression of these B-cell activation markers. The results revealed that INH-ODN 2088 increased the expression of CD86 and HLA-DR in the absence of CpG-ODN 2006 or imiquimod, and decreased only weakly the CD86 expression in their presence (Fig. 9, 10). In contrast G-modified INH-ODNs, in particular INH-ODN 21595, displayed a lower stimulatory activity and reduced significantly the CpG-ODN 2006- or imiquimod-induced expression of CD86 or HLA-DR (Fig. 9, 10). In summary, G-modification of INH-ODNs significantly improved their inhibitory potential on human B-cells stimulated via TLR7 or TLR9.

## Discussion

Our results demonstrate that the functional advantages of G-modification of INH-ODNs initially observed and described for the inhibition of murine immune cells [22] are fully applicable for human cells. In particular, G-modification largely reduced the self-stimulatory activity at high dose of INH-ODNs for human B-cells and as a consequence improved their inhibitory efficacy for this cell type. Importantly, the efficient ability of unmodified INH-ODNs to impair secretion of type I interferons by human PBMCs was not reduced by G-modification. Although G-modification was initially introduced to prevent the formation of G4-stacks in order to create INH-ODNs with a predictable pharmacological behavior, it is of particular relevance for their therapeutic potential that the inhibitory function was also increased. Additionally, we found no evidence that INH-ODNs were toxic to human PBMCs up to a concentration of 10 μM.

The almost identical inhibition pattern of this series of INH-ODNs observed with murine and human immune cells suggests that these INH-ODNs interact with identical and presumably conserved molecules. In a recent study the inhibitory potential of several INH-ODNs inhibiting TLR9 was compared for murine B-cells and the human Namalwa B-cell line as well as HEK293 cells transfected with human TLR9 [29]. The results revealed that the inhibitory strength of most INH-ODNs tested was similar for human and murine cells [29]. Our results confirm these findings for TLR9 inhibition and extend them to TLR7 inhibition, as we show here that our set of INH-ODNs induced in addition a similar inhibition pattern in murine und human B-cells stimulated via TLR7 [22].

Despite these similarities the mechanism of inhibition of TLR9-responses remains unclear. One study concluded that INH-ODN did not interfere with the uptake of CpG-ODN. Thus, G-ODN, an INH-ODN with the TLR9 inhibition motif CC(T)XXX_3–5_GGG, did not impair uptake of stimulatory CpG-ODN 1668 [30]. Similar findings were reported by Stunz et al [31]. Whether this can be extrapolated to other classes of INH-ODNs remains to be seen. It was also demonstrated that inhibitory strength and binding avidity of INH-ODNs to TLR9 did not correlate [29]. Therefore, the conserved molecule which is shared between human and mouse may not be TLR9. Instead, it was suggested that UNC93B1, which translocates TLR3, 7 and 9 from the endoplasmic reticulum to endolysosomes [32,33], might be the target for INH-ODNs [29]. However, CpG-ODN and INH-ODN bind with similar avidities to TLR9 [34], and thus a competitive mechanism for binding to TLR9 was also proposed. TLR9 is proteolytically converted after transfer from the endoplasmic reticulum to endolysosomes [35,36]. The generated C-terminal fragment of TLR9 appears to be crucial for signaling. CpG-ODN like 1826 and INH-ODNs like 2088 bound to this fragment with higher affinity than to full length TLR9 [37]. Interestingly, INH-ODN 2088 blocked the binding of CpG-ODN 1826 to the C-terminal fragment of TLR9 in RAW macrophages and impaired secretion of TNF [37]. Thus, INH-ODNs like 2088 presumably impair TLR9-induced signaling induced by CpG-ODNs by competitive binding to the C-terminal fragment of TLR9. So far similar data are not available for human TLR9.

Like TLR9, TLR7 is cleaved within its ectodomain in endolysosomes of RAW cells [35]. Based on this similarity, TLR7-INH-ODNs might also interact with the C-terminal fragment of TLR7 and by that compete with TLR7 agonists. However, sequence motifs of TLR7-INH-ODNs are less well defined. Comparison of the sequences of one set of TLR7-INH-ODNs revealed that the minimal motif required to inhibit IL-6 release by murine spleen cells stimulated with R848 was “TGC” at the 5’ end of the INH-ODN [4]. Others found that TLR7 inhibition by INH-ODNs of murine B-cells, RAW macrophages and bone marrow-derived plasmacytoid dendritic cells was sequence-independent but backbone-dependent [38]. Similarly, R848-stimulated HEK293 cells, which were stably transfected with TLR7, were impaired by INH-ODNs in a sequence-independent fashion [39]. Based on these clear sequence discrepancies between TLR7- and TLR9-INH-ODNs the mechanism of TLR7-inhibition might also be different.

So far we have not tested the activity of G-modified INH-ODNs to block human TLR8-mediated responses. Since TLR8 polymorphisms were associated with SLE, further work is required to explore the inhibitory potential of this set of INH-ODNs for human TLR8 [40].

It was postulated that G4 stacks might support the inhibitory activity of INH-ODNs by forming larger structures which may sterically hinder CpG-ODNs to interact with C-terminal TLR9 [37]. As shown here our results do not support this notion, since G-modified INH-ODNs were more effective inhibitors for TLR7- and TLR9-mediated responses. This finding was confirmed by a recent study which showed that boiling and rapid cooling of INH-ODNs like 2114 or 4347, which contained quadruple Gs and formed complex structures, reduced aggregation and improved their potency to inhibit CD86-expression of Namalwa B-cells which were stimulated with the CpG-ODN 2059 [41]. Moreover, it was shown that INH-ODN 2088 in its monomeric but not G-tetrad form inhibited IL-6 release by human B-cells stimulated with the CpG-ODN 1018 [42]. Thus, monomeric INH-ODNs are obviously more effective inhibitors than INH-ODNs with complex structures.

The most efficient of our INH-ODNs impaired IFN-α release of PBMCs at concentrations of 10 nM while IL-6 release by PBMCs and B-cells was inhibited by concentrations of 100 nM. Further optimization of INH-ODNs by 5’ extensions with sequences like “TCCTA”, “TCGTA”, “TAATA” and “CCTA” was described [29]. These changes improved the inhibitory efficacy of TLR9-specific INH-ODNs with a phosphorothioate-backbone for human cells several fold. Combination of these 5’ extensions with G-modification might further improve their inhibitory potency.

The relevance of G-modification to increase the inhibitory potential of INH-ODNs depended on the immune response analyzed. Thus, unmodified INH-ODN 2088, which was used in many different studies [29,31,37,42], was almost as efficient as G-modified INH-ODNs to impair IFN-α release by human PBMCs (Fig. 3, 4) as well as murine bone marrow-derived pDC [22]. In contrast INH-ODN 2088 failed to prevent IL-6 release induced via TLR9 from human PBMCs (Fig. 5) or B-cells (Fig. 7A, B). Although INH-ODN 2088 impeded IL-6 release by TLR7-triggered human PBMCs (Fig. 6) or B-cells (Fig. 8) it was significantly less effective compared to its G-modified variants. Similar observations were described for murine B-cells from wild type and lupus-prone mice [22]. The very low activity of INH-ODN 2088 to impair TLR9-mediated IL-6 release by human PBMCs was even more remarkable as stimulation of these cells to secrete IL-6 via TLR9 was also weak compared to the TLR7-mediated response (Fig. 1A). This difference was observed with two different TLR7- or TLR9-agonists. In part the failure of INH-ODN 2088 to impair B-cell responses can be explained by its ability to stimulate this cell type by itself. However, this phenomenon was only observed with high doses of INH-ODN 2088 and therefore other, yet unknown, mechanisms are relevant.

Patients suffering from SLE are currently treated with corticosteroids in the early phase of disease, a combination with cytotoxic agents like cyclophosphamide or mycophenolate mofetil in this phase is not beneficial [43]. Combination therapy is required, however, in cases of progressive kidney disease and more frequent lupus flares [44]. Despite this extensive immunosuppression complete remission of kidney disease is rare. This may be explained by the fact that corticosteroids failed to prevent NF-κB activation by pDCs stimulated via TLR7 and TLR9 [20]. Consequently, the secretion of IFN-α was not suppressed. Since the INH-ODNs tested here suppressed IFN-α-release by human and murine cells (Fig. 3, 4) and impaired activation of NF-κB [22], treatment of SLE patients with INH-ODNs could potentially replace therapy with corticosteroids in patients not responding to the latter. In this regard the G-modified INH-ODN 24888, which is one of the most effective INH-ODNs tested here, was significantly more effective *in vivo* than the unmodified INH-ODN 2088 and suppressed systemic IL-12p40 levels even via mucosal application [22]. Moreover, it was reasonably stable *in vivo* as pretreatment of mice for 12h still prevented systemic 12p40 levels after challenge with CpG-DNA 1826 (unpublished own observation). Regarding human cells, G-modified INH-ODN 21595 shows the best results inhibiting TLR7 and TLR9 mediated signaling and might have the most therapeutic potential as a new treatment option for SLE.

## References

[1] Baccala R, Hoebe K, Kono DH, Beutler B, Theofilopoulos AN (2007) TLR-dependent and TLR-independent pathways of type I interferon induction in systemic autoimmunity. Nat Med 13: 543–551. 17479100 10.1038/nm1590

[2] Yoshida H, Okabe Y, Kawane K, Fukuyama H, Nagata S (2005) Lethal anemia caused by interferon-beta produced in mouse embryos carrying undigested DNA. Nat Immunol 6: 49–56. 15568025 10.1038/ni1146

[3] Kono DH, Baccala R, Theofilopoulos AN (2013) TLRs and interferons: a central paradigm in autoimmunity. Curr Opin Immunol 25: 720–727. 10.1016/j.coi.2013.10.006 24246388 PMC4309276

[4] Barrat FJ, Meeker T, Gregorio J, Chan JH, Uematsu S, et al (2005) Nucleic acids of mammalian origin can act as endogenous ligands for Toll-like receptors and may promote systemic lupus erythematosus. J Exp Med 202: 1131–1139. 16230478 10.1084/jem.20050914 PMC2213213

[5] Leadbetter EA, Rifkin IR, Hohlbaum AM, Beaudette BC, Shlomchik MJ, et al (2002) Chromatin-IgG complexes activate B cells by dual engagement of IgM and Toll-like receptors. Nature 416: 603–607. 11948342 10.1038/416603a

[6] Kawasaki T, Kawai T, Akira S (2011) Recognition of nucleic acids by pattern-recognition receptors and its relevance in autoimmunity. Immunol Rev 243: 61–73. 10.1111/j.1600-065X.2011.01048.x 21884167 PMC7165622

[7] Christensen SR, Shupe J, Nickerson K, Kashgarian M, Flavell RA, et al (2006) Toll-like receptor 7 and TLR9 dictate autoantibody specificity and have opposing inflammatory and regulatory roles in a murine model of lupus. Immunity 25: 417–428. 16973389 10.1016/j.immuni.2006.07.013

[8] Pisitkun P, Deane JA, Difilippantonio MJ, Tarasenko T, Satterthwaite AB, et al (2006) Autoreactive B cell responses to RNA-related antigens due to TLR7 gene duplication. Science 312: 1669–1672. 16709748 10.1126/science.1124978

[9] Subramanian S, Tus K, Li QZ, Wang A, Tian XH, et al (2006) A Tlr7 translocation accelerates systemic autoimmunity in murine lupus. Proc Natl Acad Sci U S A 103: 9970–9975. 16777955 10.1073/pnas.0603912103 PMC1502563

[10] Fukui R, Saitoh S, Kanno A, Onji M, Shibata T, et al (2011) Unc93B1 restricts systemic lethal inflammation by orchestrating Toll-like receptor 7 and 9 trafficking. Immunity 35: 69–81. 10.1016/j.immuni.2011.05.010 21683627

[11] Sasai M, Iwasaki A (2011) Love triangle between Unc93B1, TLR7, and TLR9 prevents fatal attraction. Immunity 35: 3–5. 10.1016/j.immuni.2011.07.006 21777792 PMC3143494

[12] Koh YT, Scatizzi JC, Gahan JD, Lawson BR, Baccala R, et al (2013) Role of nucleic acid-sensing TLRs in diverse autoantibody specificities and anti-nuclear antibody-producing B cells. J Immunol 190: 4982–4990. 10.4049/jimmunol.1202986 23589617 PMC3729324

[13] Demaria O, Pagni PP, Traub S, de Gassart A, Branzk N, et al (2010) TLR8 deficiency leads to autoimmunity in mice. J Clin Invest 120: 3651–3662. 10.1172/JCI42081 20811154 PMC2947223

[14] Vollmer J, Tluk S, Schmitz C, Hamm S, Jurk M, et al (2005) Immune stimulation mediated by autoantigen binding sites within small nuclear RNAs involves Toll-like receptors 7 and 8. J Exp Med 202: 1575–1585. 16330816 10.1084/jem.20051696 PMC2213330

[15] Marshak-Rothstein A (2006) Toll-like receptors in systemic autoimmune disease. Nat Rev Immunol 6: 823–835. 17063184 10.1038/nri1957 PMC7097510

[16] Savarese E, Chae OW, Trowitzsch S, Weber G, Kastner B, et al (2006) U1 small nuclear ribonucleoprotein immune complexes induce type I interferon in plasmacytoid dendritic cells through TLR7. Blood 107: 3229–3234. 16368889 10.1182/blood-2005-07-2650

[17] Yanai H, Ban T, Wang Z, Choi MK, Kawamura T, et al (2009) HMGB proteins function as universal sentinels for nucleic-acid-mediated innate immune responses. Nature 462: 99–103. 10.1038/nature08512 19890330

[18] Lande R, Gregorio J, Facchinetti V, Chatterjee B, Wang YH, et al (2007) Plasmacytoid dendritic cells sense self-DNA coupled with antimicrobial peptide. Nature 449: 564–569. 17873860 10.1038/nature06116

[19] Means TK, Latz E, Hayashi F, Murali MR, Golenbock DT, et al (2005) Human lupus autoantibody-DNA complexes activate DCs through cooperation of CD32 and TLR9. J Clin Invest 115: 407–417. 15668740 10.1172/JCI23025 PMC544604

[20] Guiducci C, Gong M, Xu Z, Gill M, Chaussabel D, et al (2010) TLR recognition of self nucleic acids hampers glucocorticoid activity in lupus. Nature 465: 937–941. 10.1038/nature09102 20559388 PMC2964153

[21] Lenert PS (2010) Classification, mechanisms of action, and therapeutic applications of inhibitory oligonucleotides for Toll-like receptors (TLR) 7 and 9. Mediators Inflamm 2010: 986596 10.1155/2010/986596 20490286 PMC2873634

[22] Römmler F, Jurk M, Uhlmann E, Hammel M, Waldhuber A, et al (2013) Guanine modification of inhibitory oligonucleotides potentiates their suppressive function. J Immunol 191: 3240–3253. 10.4049/jimmunol.1300706 23966630

[23] Forsbach A, Nemorin JG, Montino C, Muller C, Samulowitz U, et al (2008) Identification of RNA sequence motifs stimulating sequence-specific TLR8-dependent immune responses. J Immunol 180: 3729–3738. 18322178 10.4049/jimmunol.180.6.3729

[24] Hartmann G, Krieg AM (2000) Mechanism and function of a newly identified CpG DNA motif in human primary B cells. J Immunol 164: 944–953. 10623843 10.4049/jimmunol.164.2.944

[25] Krug A, Rothenfusser S, Hornung V, Jahrsdorfer B, Blackwell S, et al (2001) Identification of CpG oligonucleotide sequences with high induction of IFN-alpha/beta in plasmacytoid dendritic cells. Eur J Immunol 31: 2154–2163. 11449369 10.1002/1521-4141(200107)31:7<2154::aid-immu2154>3.0.co;2-u

[26] Crouch SP, Kozlowski R, Slater KJ, Fletcher J (1993) The use of ATP bioluminescence as a measure of cell proliferation and cytotoxicity. J Immunol Methods 160: 81–88. 7680699 10.1016/0022-1759(93)90011-u

[27] Siegal FP, Kadowaki N, Shodell M, Fitzgerald-Bocarsly PA, Shah K, et al (1999) The nature of the principal type 1 interferon-producing cells in human blood. Science 284: 1835–1837. 10364556 10.1126/science.284.5421.1835

[28] Hornung V, Rothenfusser S, Britsch S, Krug A, Jahrsdorfer B, et al (2002) Quantitative expression of toll-like receptor 1–10 mRNA in cellular subsets of human peripheral blood mononuclear cells and sensitivity to CpG oligodeoxynucleotides. J Immunol 168: 4531–4537. 11970999 10.4049/jimmunol.168.9.4531

[29] Ashman RF, Goeken JA, Latz E, Lenert P (2011) Optimal oligonucleotide sequences for TLR9 inhibitory activity in human cells: lack of correlation with TLR9 binding. Int Immunol 23: 203–214. 10.1093/intimm/dxq473 21393636 PMC3053407

[30] Peter M, Bode K, Lipford GB, Eberle F, Heeg K, et al (2008) Characterization of suppressive oligodeoxynucleotides that inhibit Toll-like receptor-9-mediated activation of innate immunity. Immunology 123: 118–128. 17961163 10.1111/j.1365-2567.2007.02718.x PMC2433270

[31] Stunz LL, Lenert P, Peckham D, Yi AK, Haxhinasto S, et al (2002) Inhibitory oligonucleotides specifically block effects of stimulatory CpG oligonucleotides in B cells. Eur J Immunol 32: 1212–1222. 11981808 10.1002/1521-4141(200205)32:5<1212::AID-IMMU1212>3.0.CO;2-D

[32] Kim YM, Brinkmann MM, Paquet ME, Ploegh HL (2008) UNC93B1 delivers nucleotide-sensing toll-like receptors to endolysosomes. Nature 452: 234–238. 10.1038/nature06726 18305481

[33] Brinkmann MM, Spooner E, Hoebe K, Beutler B, Ploegh HL, et al (2007) The interaction between the ER membrane protein UNC93B and TLR3, 7, and 9 is crucial for TLR signaling. J Cell Biol 177: 265–275. 17452530 10.1083/jcb.200612056 PMC2064135

[34] Latz E, Verma A, Visintin A, Gong M, Sirois CM, et al (2007) Ligand-induced conformational changes allosterically activate Toll-like receptor 9. Nat Immunol 8: 772–779. 17572678 10.1038/ni1479

[35] Ewald SE, Lee BL, Lau L, Wickliffe KE, Shi GP, et al (2008) The ectodomain of Toll-like receptor 9 is cleaved to generate a functional receptor. Nature 456: 658–662. 10.1038/nature07405 18820679 PMC2596276

[36] Park B, Brinkmann MM, Spooner E, Lee CC, Kim YM, et al (2008) Proteolytic cleavage in an endolysosomal compartment is required for activation of Toll-like receptor 9. Nat Immunol 9: 1407–1414. 10.1038/ni.1669 18931679 PMC2735466

[37] Avalos AM, Ploegh HL (2011) Competition by inhibitory oligonucleotides prevents binding of CpG to C-terminal TLR9. Eur J Immunol 41: 2820–2827. 10.1002/eji.201141563 21766476 PMC3746339

[38] Lenert P, Yasuda K, Busconi L, Nelson P, Fleenor C, et al (2009) DNA-like class R inhibitory oligonucleotides (INH-ODNs) preferentially block autoantigen-induced B-cell and dendritic cell activation in vitro and autoantibody production in lupus-prone MRL-Fas(lpr/lpr) mice in vivo. Arthritis Res Ther 11: R79 10.1186/ar2710 19476613 PMC2714127

[39] Jurk M, Kritzler A, Schulte B, Tluk S, Schetter C, et al (2006) Modulating responsiveness of human TLR7 and 8 to small molecule ligands with T-rich phosphorothiate oligodeoxynucleotides. Eur J Immunol 36: 1815–1826. 16783850 10.1002/eji.200535806

[40] Armstrong DL, Reiff A, Myones BL, Quismorio FP Jr, Klein-Gitelman M, et al (2009) Identification of new SLE-associated genes with a two-step Bayesian study design. Genes Immun 10: 446–456. 10.1038/gene.2009.38 19440200 PMC3434884

[41] Ashman RF, Goeken JA, Lenert PS (2011) Aggregation and secondary loop structure of oligonucleotides do not determine their ability to inhibit TLR9. Int Immunopharmacol 11: 1032–1037. 10.1016/j.intimp.2011.02.023 21376154 PMC3119753

[42] Duramad O, Fearon KL, Chang B, Chan JH, Gregorio J, et al (2005) Inhibitors of TLR-9 act on multiple cell subsets in mouse and man in vitro and prevent death in vivo from systemic inflammation. J Immunol 174: 5193–5200. 15843514 10.4049/jimmunol.174.9.5193

[43] Rovin BH, Parikh SV (2014) Lupus Nephritis: The Evolving Role of Novel Therapeutics. Am J Kidney Dis. 10.1053/j.ajkd.2013.11.023 PMC4159074 24411715

[44] Austin HA 3rd, Klippel JH, Balow JE, le Riche NG, Steinberg AD, et al (1986) Therapy of lupus nephritis. Controlled trial of prednisone and cytotoxic drugs. N Engl J Med 314: 614–619. 3511372 10.1056/NEJM198603063141004

